# Control of Canalization and Evolvability by Hsp90

**DOI:** 10.1371/journal.pone.0000075

**Published:** 2006-12-20

**Authors:** Claire C. Milton, Christina M. Ulane, Suzannah Rutherford

**Affiliations:** 1 Center for Environmental Stress and Adaptation Research, University of Melbourne, Melbourne, Australia; 2 Division of Basic Sciences, Fred Hutchinson Cancer Research Center, Seattle, Washington, United States of America; Max Planck Institute of Molecular Cell Biology and Genetics, Germany

## Abstract

Partial reduction of Hsp90 increases expression of morphological novelty in qualitative traits of Drosophila and Arabidopsis, but the extent to which the Hsp90 chaperone also controls smaller and more likely adaptive changes in natural quantitative traits has been unclear. To determine the effect of Hsp90 on quantitative trait variability we deconstructed genetic, stochastic and environmental components of variation in Drosophila wing and bristle traits of genetically matched flies, differing only by Hsp90 loss-of-function or wild-type alleles. Unexpectedly, Hsp90 buffering was remarkably specific to certain normally invariant and highly discrete quantitative traits. Like the qualitative trait phenotypes controlled by Hsp90, highly discrete quantitative traits such as scutellor and thoracic bristle number are threshold traits. When tested across genotypes sampled from a wild population or in laboratory strains, the sensitivity of these traits to many types of variation was coordinately controlled, while continuously variable bristle types and wing size, and critically invariant left-right wing asymmetry, remained relatively unaffected. Although increased environmental variation and developmental noise would impede many types of selection response, in replicate populations in which Hsp90 was specifically impaired, heritability and ‘extrinsic evolvability’, the expected response to selection, were also markedly increased. However, despite the overall buffering effect of Hsp90 on variation in populations, for any particular individual or genotype in which Hsp90 was impaired, the size and direction of its effects were unpredictable. The trait and genetic-background dependence of Hsp90 effects and its remarkable bias toward invariant or canalized traits support the idea that traits evolve independent and trait-specific mechanisms of canalization and evolvability through their evolution of non-linearity and thresholds. Highly non-linear responses would buffer variation in Hsp90-dependent signaling over a wide range, while over a narrow range of signaling near trait thresholds become more variable with increasing probability of triggering all-or-none developmental responses.

## Introduction

The Hsp90 stress response protein is an ancient, abundant and nearly ubiquitous protein chaperone that supports an essential group of conserved signal transduction proteins in nearly every organism and cell type examined [1]–[3]. A minimal level of Hsp90 is required for cell viability, however signaling by Hsp90 substrates is sensitive to much smaller reductions of the chaperone—in heterozygous mutants that are fully viable and apparently normal, signaling through numerous Hsp90 target pathways is already reduced. For example, in highly inbred flies, heterozygous Hsp90 mutants dominantly enhance heterozygous or partial function mutations of Hsp90 targets and other genes in Hsp90-dependent pathways, revealing previously unexpressed developmental phenotypes [4], [5]. In outbred populations, partial reduction of Hsp90 reveals natural genetic variation for many and varied developmental phenotypes [6], [7]. The expression of Hsp90-buffered phenotypes in wild populations or laboratory strains of Drosophila likely depends on the chance segregation of multiple, previously undetected polymorphisms in a few Hsp90 targets and multiple non-target genes with indirect effects on Hsp90-dependent pathways (SR; in preparation).

A balance between Hsp90 buffering and the expression of morphological traits is most likely moderated in nature by environmental stress. Stress-damaged proteins titrate Hsp90 from signal transduction proteins; despite transcriptional induction of Hsp90 and other chaperones by the heat shock response, it is believed that severe stresses in nature can temporarily overwhelm the Hsp90 chaperone system [2], [8]. Indeed, across diverse species, Hsp90 buffers widespread morphological variation or controls characteristic developmental transitions, for example altered morphologies in laboratory and wild populations of Drosophila [6] and Arabidopsis [7], cortical patterning in Tetrahymena [9], [10], developmental progression in fungi [11], [12], and metamorphosis in Leishmania parasites [13] and Ascidian embryos [14]. Although there is evidence supporting the occurrence of phenotypes controlled by Hsp90 during laboratory heat shock [9], [10] and by associated chaperones during heat stress in nature [8], the relevance of the Hsp90 for evolution remains challenged by allegations that morphological changes controlled by Hsp90 are unequivocally “monstrous”, and that Hsp90-buffered variation is “unconditionally deleterious” [7], [15]–[18]. The extent to which the Hsp90 chaperone also controls smaller and more-likely adaptive changes in natural quantitative traits has been unclear [19], [20].

Heritable variation with small effects on quantitative traits is widely believed to be the usual substrate of most evolutionary change. In Drosophila, the number and position of mechanosensory bristles, and wing size and shape are most likely optimized by natural selection [21]–[23]. While many quantitative traits are normally variable, the stereotypical placement and number of scutellar (SC) and thoracic (TH) bristles is conserved across fly species and nearly invariant within and between populations [24]. As a result of their intra- and inter-specific invariance, SC and TH bristle numbers have been extensively studied models of canalization [25], [26], a developmental robustness to variation believed to have evolved through strong stabilizing selection against genetic and/or environmental perturbations of an optimum phenotype [27], [28]. Current models of canalization suggest that genetic buffering occurs as a by-product of environmental canalization, which should evolve under stronger and more consistent selective pressure [18], [29]. These models predict that genetic and environmental canalization would therefore share common mechanism(s) [18], [29]–[31], but this idea has not been tested for any canalized trait and population. Since Hsp90 mediates the impacts of genetic variation and environmental stress, it is a logical candidate for a “common mechanism of canalization” [18]. We previously reported that Hsp90 had no effect on measures of environmental canalization of several normally variable quantitative traits while at the same time, across the same set of flies, Hsp90 did control morphological variation of qualitative features specific to particular strain backgrounds, measures of genetic canalization [19]. This suggested that genetic and environmental buffering by Hsp90 are separable. The dissociation of its role buffering genetic variation from the more obvious role of Hsp90 as a stress-response protein buffering environmental variation calls into question the mechanism by which genetic canalization evolves.

Here we dissect the relationship between Hsp90 buffering, genetic and non-genetic components of morphological variation, canalization and evolvability in a large set of quantitative traits across a controlled population of flies with well-defined genetic backgrounds. We now report that Hsp90 also had little effect on the phenotypic variability or evolvability of the normally variable wing and bristle traits. Unexpectedly, Hsp90 effects were remarkably specific to the most invariant quantitative traits showing that whether Hsp90-buffered traits are quantitative or qualitative, they are threshold traits with a continuous underlying distribution of genetic and environmental effects (liability) determining the probability that they would be variably expressed. Consistent with theoretical predictions for the evolution of genetic canalization, Hsp90 simultaneously buffered components of genetic, stochastic and environmental variation. Under low Hsp90, predicted responses to selection of Hsp90-buffered traits increased with increased expression of variation. We conclude that during unpredictable changes and stressful environmental changes in nature, partial reductions of Hsp90 would destabilize previously invariable or canalized threshold traits and transiently provide a random expression of previously invariable quantitative trait phenotypes upon which selection could act.

## Results

### Experimental partition of genetic and non-genetic effects

**Figure 1:**
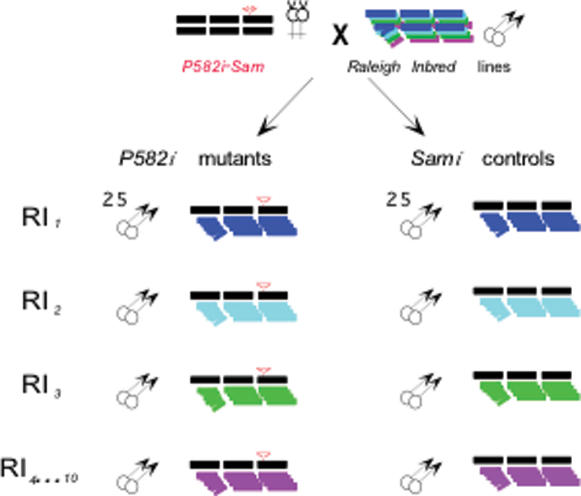
Experimental design partitions genetic and purely-environmental components of variation. Females heterozygous for *P582i* (*Sam1;Sam2;P582i/Sami*; left) were crossed to males from each of 9 highly inbred (95% homozygous) *RI* line backgrounds (different colors) to create matched “clones” of Hsp90 mutant and control male progeny from the same vial and maternal environments and hybrid for *Sam* and each *RI* line background.

We deconstructed the effect of Hsp90 on small variations in standard quantitative traits using genetically matched groups of F_1_ heterozygotes, differing only in whether they carried Hsp90 mutant (*P582i*) or control (*Sami*) alleles of Hsp90 on otherwise identical genetic backgrounds. Random differences in left-right symmetry, trait values among isogenic flies and genetic effects on trait means across wild backgrounds were used to parse developmental noise, micro-environmental and genetic variation. *P582* is a marked P-element disruption of Hsp90 [32], which is not viable when homozygous and likely makes no Hsp90 protein [33]. When heterozygous, *P582* strongly reduces signaling through Hsp90-dependent pathways and uncovers previously cryptic morphogenic variation [2], [6], [19], [34]. To ensure that Hsp90 mutant and control flies were isogenic, *P582* was introduced into an extremely inbred and essentially homozygous wild background, *Samarkand* (*Sam*), by over 38 generations of backcrossing, introgression, and homogenization (creating *P582i*) [19]. As shown in Figure 1, genetically identical mutant and control flies from the same maternal and vial environments were produced by crossing heterozygous *P582i* females to males from 9 highly inbred (>95% homozygous) Raleigh Inbred (*RI*) lines representing genotypes sampled at random from a wild population. To the extent that the *Sam* and *RI* lines were completely homozygous, the groups of F_1_ progeny were clones, hybrid for *Sam* and each *RI* line background, and differing only by Hsp90 allele.

To partition as cleanly as possible the effect of Hsp90 on genetic versus non-genetic variation, variation among the groups of 50 isogenic flies within each replicated genotype was used to measure the effect of Hsp90 on all sources of purely non-genetic variation (*V_e_*), and variation among the means of the *RI* line genotypes was used to measure its effect on genetic variation (*V_G_*) from all sources of heritable variation segregating in the set of 9 *RI* line backgrounds. Total phenotypic variation (*V_P_*) from the combined effects of genetic variation, environmental variation and developmental noise was derived from measurements on all 450 flies of every *RI* line genotype within each Hsp90 allele.

### Phenotypic canalization and variability

**Figure 2:**
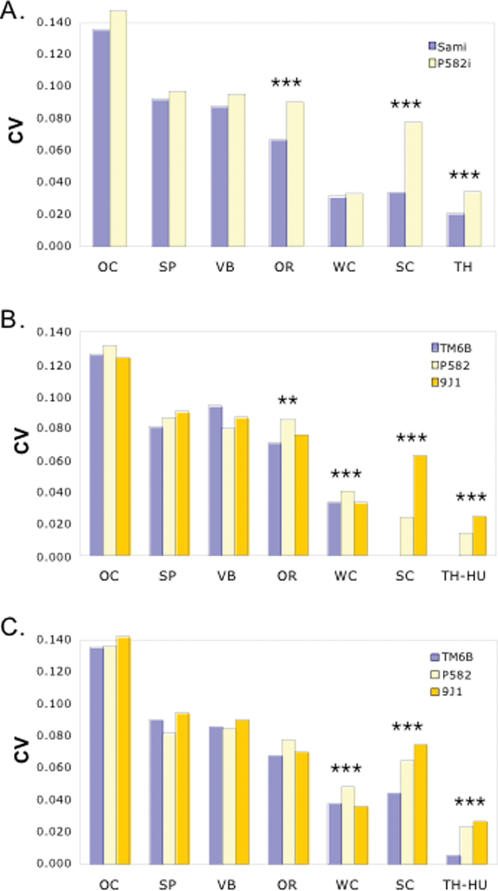
Hsp90 controlled phenotypic variation of most invariant quantitative traits. (**A**) Effects of *P582i* (yellow) and *Sami* (blue) alleles of Hsp90 in the isogenic *Sam* background on *V_P_* of mutant and control sets of 450 males across the 9 *RI* line backgrounds. (**B and C**) Effects of 3^rd^ chromosomes carrying null (*P582*), and dominant-negative (*9J1*) Hsp90 mutations or wild-type Sam alleles introgressed into the *Sam* background in mutant and control sets of 225 male or 225 female sibs. *TM6B* contains the dominant *Humeral* (*Hu*) mutation [32], which increases humeral TH bristle numbers. Therefore, for comparison of TH bristles between TM6B and the other genotypes we used TH-HU, indicating that TH was scored only for the remaining 18 non-humeral TH bristle types. All experiments were conducted under temperature, density and humidity controlled conditions. Environmental effects were further controlled by direct comparisons between flies from the same vial and maternal environments. Coefficients of variation (*CV* = standard deviation/mean) are shown to enable between-trait comparisons. Statistical tests of the significance of Hsp90 effects on phenotypic variability and *P*-values are found in Table 1 (for A) and Table 2 (for B and C).

There was a sharp distinction between the effects of Hsp90 on discrete quantitative traits, which usually do not vary between flies, and those that are naturally more variable. As shown previously, normally-variable wing and bristle traits were unaffected by Hsp90 [19]. However, Hsp90 had highly significant and reproducible effects on total phenotypic variation (*V_P_*) of scutellar (SC) and thoracic (TH) bristles (Fig. 2 and Tables 1 and 2), which are among the most invariant bristle traits in Drosophila, and have been previously studied models of canalization [25], [26]. Twice as many Hsp90-mutant flies (*P582i*) had abnormal TH bristle numbers, and the severity of the TH bristle number defects increased from deviations of at most 2 TH bristles to as many as four. Even more striking, the number of flies with aberrant SC bristle numbers increased 4 to 5-fold in the Hsp90-mutants, from 8 control flies differing by a single SC bristle and clustered in a few *RI* line backgrounds, to 35 more severely abnormal *P582i* flies found multiply in every *RI* line background.

**Table 1:**
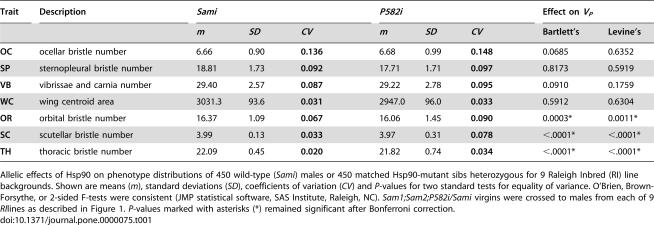
Hsp90 controlled phenotypic variability of normally invariant bristle types in isogenic males heterozygous for Hsp90 mutant (*P582i*) or control (*Sami*) alleles with otherwise identical genetic backgrounds.

Trait	Description	*Sami*			*P582i*			Effect on *V_P_*	
		*m*	*SD*	*CV*	*m*	*SD*	*CV*	Bartlett's	Levine's
**OC**	ocellar bristle number	6.66	0.90	**0.136**	6.68	0.99	**0.148**	0.0685	0.6352
**SP**	sternopleural bristle number	18.81	1.73	**0.092**	17.71	1.71	**0.097**	0.8173	0.5919
**VB**	vibrissae and carnia number	29.40	2.57	**0.087**	29.22	2.78	**0.095**	0.0910	0.1759
**WC**	wing centroid area	3031.3	93.6	**0.031**	2947.0	96.0	**0.033**	0.5912	0.6304
**OR**	orbital bristle number	16.37	1.09	**0.067**	16.06	1.45	**0.090**	0.0003*	0.0011*
**SC**	scutellar bristle number	3.99	0.13	**0.033**	3.97	0.31	**0.078**	<.0001*	<.0001*
**TH**	thoracic bristle number	22.09	0.45	**0.020**	21.82	0.74	**0.034**	<.0001*	<.0001*

Allelic effects of Hsp90 on phenotype distributions of 450 wild-type (*Sami*) males or 450 matched Hsp90-mutant sibs heterozygous for 9 Raleigh Inbred (RI) line backgrounds. Shown are means (*m*), standard deviations (*SD*), coefficients of variation (*CV*) and *P*-values for two standard tests for equality of variance. O'Brien, Brown-Forsythe, or 2-sided F-tests were consistent (JMP statistical software, SAS Institute, Raleigh, NC). *Sam1;Sam2;P582i/Sami* virgins were crossed to males from each of 9* RI*lines as described in Figure 1. *P*-values marked with asterisks (*) remained significant after Bonferroni correction.

**Table 2:**
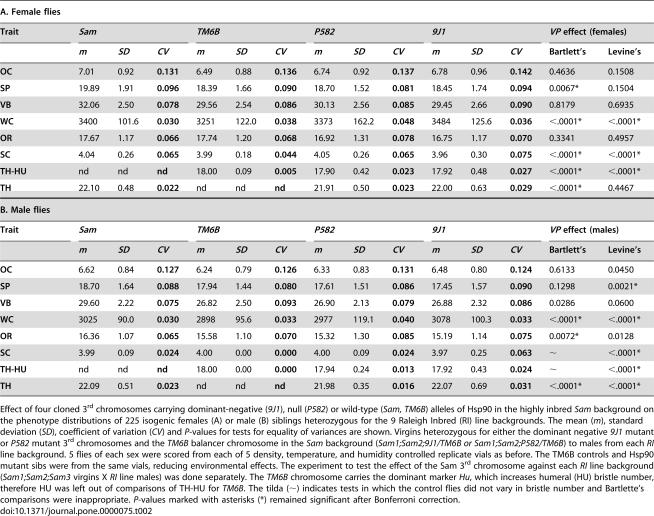
Null (*P582*) and dominant negative (*9J1*) Hsp90-mutant chromosomes increased the phenotypic variability of normally invariant bristle types in both female and male flies.

A. Female flies														
Trait	*Sam*			*TM6B*			*P582*			*9J1*			*VP* effect (females)	
	*m*	*SD*	*CV*	*m*	*SD*	*CV*	*m*	*SD*	*CV*	*m*	*SD*	*CV*	Bartlett's	Levine's
**OC**	7.01	0.92	**0.131**	6.49	0.88	**0.136**	6.74	0.92	**0.137**	6.78	0.96	**0.142**	0.4636	0.1508
**SP**	19.89	1.91	**0.096**	18.39	1.66	**0.090**	18.70	1.52	**0.081**	18.45	1.74	**0.094**	0.0067*	0.1504
**VB**	32.06	2.50	**0.078**	29.56	2.54	**0.086**	30.13	2.56	**0.085**	29.45	2.66	**0.090**	0.8179	0.6935
**WC**	3400	101.6	**0.030**	3251	122.0	**0.038**	3373	162.2	**0.048**	3484	125.6	**0.036**	<.0001*	<.0001*
**OR**	17.67	1.17	**0.066**	17.74	1.20	**0.068**	16.92	1.31	**0.078**	16.75	1.17	**0.070**	0.3341	0.4957
**SC**	4.04	0.26	**0.065**	3.99	0.18	**0.044**	4.05	0.26	**0.065**	3.96	0.30	**0.075**	<.0001*	<.0001*
**TH-HU**	nd	nd	**nd**	18.00	0.09	**0.005**	17.90	0.42	**0.023**	17.92	0.48	**0.027**	<.0001*	<.0001*
**TH**	22.10	0.48	**0.022**	nd	nd	**nd**	21.91	0.50	**0.023**	22.00	0.63	**0.029**	<.0001*	0.4467

B. Male flies														
Trait	*Sam*			*TM6B*			*P582*			*9J1*			*VP* effect (males)	
	*m*	*SD*	*CV*	*m*	*SD*	*CV*	*m*	*SD*	*CV*	*m*	*SD*	*CV*	Bartlett's	Levine's
**OC**	6.62	0.84	**0.127**	6.24	0.79	**0.126**	6.33	0.83	**0.131**	6.48	0.80	**0.124**	0.6133	0.0450
**SP**	18.70	1.64	**0.088**	17.94	1.44	**0.080**	17.61	1.51	**0.086**	17.45	1.57	**0.090**	0.1298	0.0021*
**VB**	29.60	2.22	**0.075**	26.82	2.50	**0.093**	26.90	2.13	**0.079**	26.88	2.32	**0.086**	0.0286	0.0600
**WC**	3025	90.0	**0.030**	2898	95.6	**0.033**	2977	119.1	**0.040**	3078	100.3	**0.033**	<.0001*	<.0001*
**OR**	16.36	1.07	**0.065**	15.58	1.10	**0.070**	15.32	1.30	**0.085**	15.19	1.14	**0.075**	0.0072*	0.0128
**SC**	3.99	0.09	**0.024**	4.00	0.00	**0.000**	4.00	0.09	**0.024**	3.97	0.25	**0.063**	∼	<.0001*
**TH-HU**	nd	nd	**nd**	18.00	0.00	**0.000**	17.94	0.24	**0.013**	17.92	0.43	**0.024**	∼	<.0001*
**TH**	22.09	0.51	**0.023**	nd	nd	**nd**	21.98	0.35	**0.016**	22.07	0.69	**0.031**	<.0001*	<.0001*

Effect of four cloned 3^rd^ chromosomes carrying dominant-negative (*9J1*), null (*P582*) or wild-type (*Sam, TM6B*) alleles of Hsp90 in the highly inbred *Sam* background on the phenotype distributions of 225 isogenic females (A) or male (B) siblings heterozygous for the 9 Raleigh Inbred (RI) line backgrounds. The mean (*m*), standard deviation (*SD*), coefficient of variation (*CV*) and *P*-values for tests for equality of variances are shown. Virgins heterozygous for either the dominant negative *9J1* mutant or *P582* mutant 3^rd^ chromosomes and the *TM6B* balancer chromosome in the *Sam* background (*Sam1;Sam2;9J1/TM6B or Sam1;Sam2;P582/TM6B*) to males from each* RI* line background. 5 flies of each sex were scored from each of 5 density, temperature, and humidity controlled replicate vials as before. The TM6B controls and Hsp90 mutant sibs were from the same vials, reducing environmental effects. The experiment to test the effect of the Sam 3^rd^ chromosome against each *RI* line background (*Sam1;Sam2;Sam3* virgins X *RI* line males) was done separately. The *TM6B* chromosome carries the dominant marker *Hu*, which increases humeral (HU) bristle number, therefore HU was left out of comparisons of TH-HU for *TM6B*. The tilda (∼) indicates tests in which the control flies did not vary in bristle number and Bartlette's comparisons were inappropriate. *P*-values marked with asterisks (*) remained significant after Bonferroni correction.

The specificity of Hsp90 toward the most invariant bristle traits did not depend on the particular Hsp90 allele or on the specific genetic background. When genetically outbred, wild-type flies were fed the Hsp90 inhibitor geldanamycin as 2^nd^ or 3^rd^ instar larvae, *CV*_SC_ increased from 0, e.g. no abnormal flies among 100 flies tested, to 0.025 (*P* = 0.043 by Levine's test) and *CV*_TH_ increased from 0.010 to 0.026 (*P*<0.0001). Phenotypic variation in the other, more variable traits was unaffected by geldanamycin [19]. In a third experiment (Table 2), variation in SC, TH and OR was similarly increased by either loss-of-function (*P582*) or dominant negative (*9J1*) alleles of Hsp90 in the context of cloned 3^rd^ chromosomes compared with wild-type alleles of Hsp90 on *Sam* or balancer (*TM6B*) 3^rd^ chromosomes. Across the set of *RI* lines in the inbred *Sam* background flies had genotypes differing by the Hsp90 allele and third chromosomes (more than 1/3 of the genome). Interestingly, as reported for morphological traits [6], [7], the specific effect of Hsp90 on any inbred *RI* line depended on genetic background (highly significant Hsp90 X *RI* line interactions; Table 3). As has been generally proposed for canalized traits [35], these studies suggest that Hsp90 controls variability—the *capacity* to vary [36], through multiple genetic interactions (molecular epistasis) with its chaperone targets and through them, other polymorphic genes.

**Table 3:**
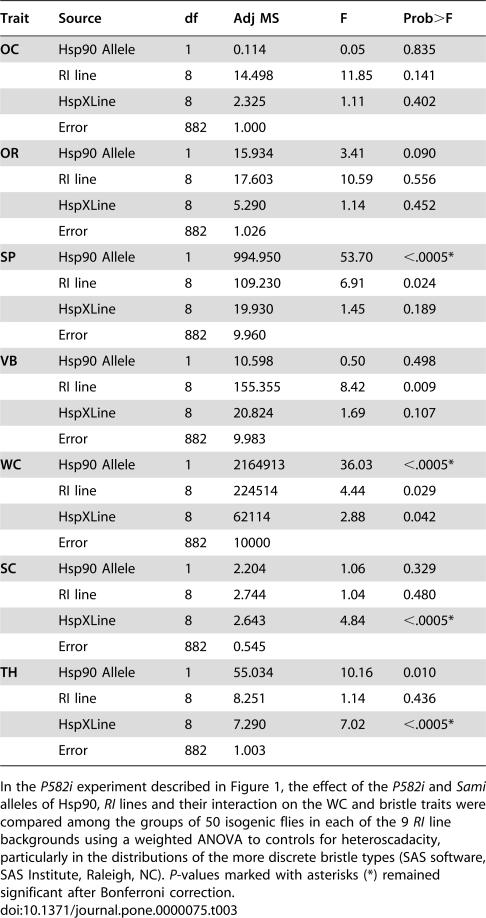
The effect of Hsp90 on variation in normally invariant bristle types depended on the *RI*-line genetic background.

Trait	Source	df	Adj MS	F	Prob>F
**OC**	Hsp90 Allele	1	0.114	0.05	0.835
	RI line	8	14.498	11.85	0.141
	HspXLine	8	2.325	1.11	0.402
	Error	882	1.000		
**OR**	Hsp90 Allele	1	15.934	3.41	0.090
	RI line	8	17.603	10.59	0.556
	HspXLine	8	5.290	1.14	0.452
	Error	882	1.026		
**SP**	Hsp90 Allele	1	994.950	53.70	<.0005*
	RI line	8	109.230	6.91	0.024
	HspXLine	8	19.930	1.45	0.189
	Error	882	9.960		
**VB**	Hsp90 Allele	1	10.598	0.50	0.498
	RI line	8	155.355	8.42	0.009
	HspXLine	8	20.824	1.69	0.107
	Error	882	9.983		
**WC**	Hsp90 Allele	1	2164913	36.03	<.0005*
	RI line	8	224514	4.44	0.029
	HspXLine	8	62114	2.88	0.042
	Error	882	10000		
**SC**	Hsp90 Allele	1	2.204	1.06	0.329
	RI line	8	2.744	1.04	0.480
	HspXLine	8	2.643	4.84	<.0005*
	Error	882	0.545		
**TH**	Hsp90 Allele	1	55.034	10.16	0.010
	RI line	8	8.251	1.14	0.436
	HspXLine	8	7.290	7.02	<.0005*
	Error	882	1.003		

In the *P582i* experiment described in Figure 1, the effect of the *P582i* and *Sami* alleles of Hsp90, *RI* lines and their interaction on the WC and bristle traits were compared among the groups of 50 isogenic flies in each of the 9 *RI* line backgrounds using a weighted ANOVA to controls for heteroscadacity, particularly in the distributions of the more discrete bristle types (SAS software, SAS Institute, Raleigh, NC). *P*-values marked with asterisks (*) remained significant after Bonferroni correction.

### Developmental noise and micro-environmental variation

To ask whether Hsp90 also controlled components of environmental canalization, we measured its effect on two sources of non-genetic variation in the same set of wing and bristle traits in the same groups of *P582i* mutant and *Sami* control flies. Developmental noise (*V_e_ within*) results in stochastic perturbations of identical left and right features as they develop within single flies of the same genotype and environment. To estimate *V_e_ within* for each *RI* line, we averaged left-right measures of developmental noise [(L−R)/(L+R/2)] over the groups of 50 isogenic flies within each *RI* line background (mean fluctuating asymmetry [37], [38]). Purely environmental variation (*V_e_ among* [39]), unique to the micro-environments of individual flies, was measured directly as variation in each trait (L+R) among the same groups of flies.

**Figure 3:**
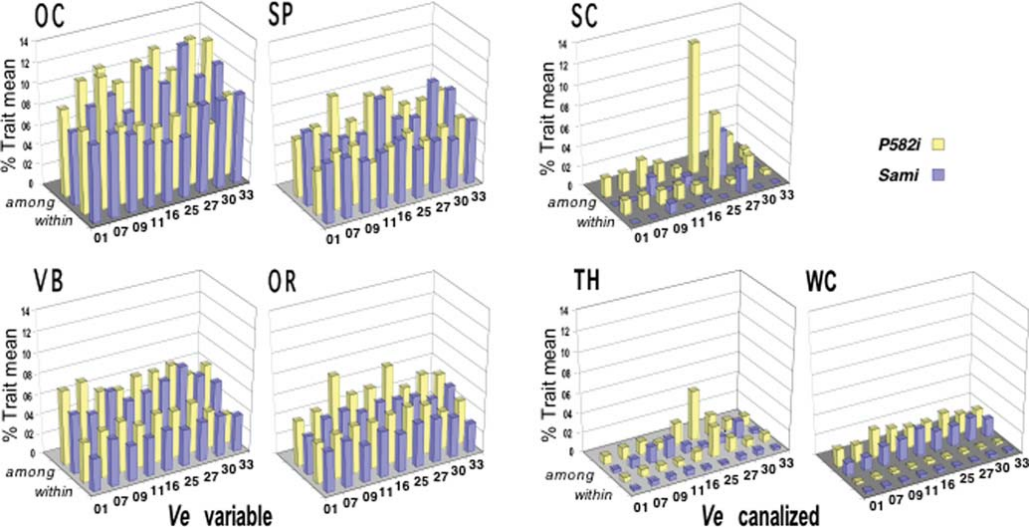
Hsp90 controlled environmental variation specific to previously invariant or canalized traits. Comparison of mean-normalized components of purely-environmental variation (z-axis) across the *RI* line backgrounds (x-axis). Developmental stability was calculated as the averaged (unsigned) deviations of left and right from the mean, i.e. (L+R)/2, within each individual (*V_e_ within*) or on the averaged (unsigned) deviations of each individual (L+R) from their clone means for each *RI* line genotype (*V_e_ among*). There was generally no effect of Hsp90 allele (*Sami*, blue or *P582i*, yellow) on the variable traits, and a highly significant effect on either measure of *V_e_* for canalized bristle traits but not wing area, which was highly canalized independent of Hsp90 (Table 4).

When either measure of *V_e_* was normalized by the mean trait value for each *RI* line background, the difference between the more variable and the invariant traits was striking (Fig. 3; compare left to right). While *V_e_ within* or *V_e_ among* was up to 10% of the mean of the normally variable OC, SP and VB bristle numbers, they were at least 10–100 fold lower for the most invariant traits (SC, TH, WC). Interestingly, either measure of *V_e_* for WC was tightly controlled independent of Hsp90, while both forms of non-genetic variation in SC and TH were sharply increased in the Hsp90 mutants (*P582i*; Table 4). For SC and TH, Hsp90 simultaneously buffered quantitative variation specific to genetic backgrounds and variation from non-genetic (stochastic) developmental and environmental effects, suggesting that for particular traits, Hsp90 buffering provides a shared mechanism of genetic and environmental canalization. However, the curious trait-specificity and genetic-background dependence of Hsp90 effects is inconsistent with Hsp90 having evolved as a “canalization gene”, but suggests instead that traits evolve (or simply acquire, unselected), trait-specific and independent mechanisms buffering genetic and environmental variation. By promoting the activity of many developmental regulators, Hsp90 may simply be positioned as a passive regulator of interactions between multiple developmental pathways and competing genetic and environmental effects.

**Table 4:**
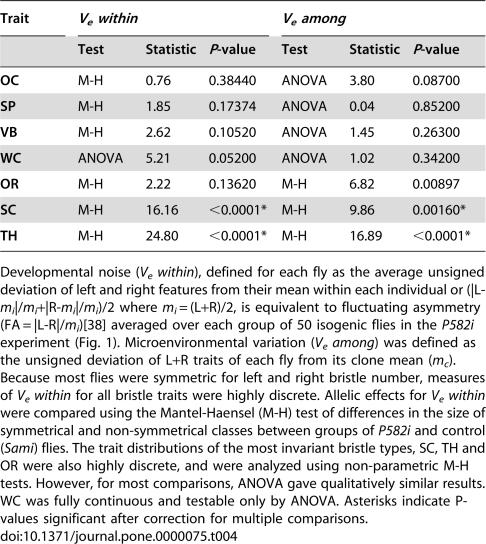
Hsp90 controlled developmental noise and micro-environmental variation of previously invariant bristle types against.

Trait	*V_e_ within*			*V_e_ among*		
	Test	Statistic	*P*-value	Test	Statistic	*P*-value
**OC**	M-H	0.76	0.38440	ANOVA	3.80	0.08700
**SP**	M-H	1.85	0.17374	ANOVA	0.04	0.85200
**VB**	M-H	2.62	0.10520	ANOVA	1.45	0.26300
**WC**	ANOVA	5.21	0.05200	ANOVA	1.02	0.34200
**OR**	M-H	2.22	0.13620	M-H	6.82	0.00897
**SC**	M-H	16.16	<0.0001*	M-H	9.86	0.00160*
**TH**	M-H	24.80	<0.0001*	M-H	16.89	<0.0001*

Developmental noise (*V_e_ within*), defined for each fly as the average unsigned deviation of left and right features from their mean within each individual or (|L-*m_i_*|/*m_i_*+|R-*m_i_*|/*m_i_*)/2 where *m_i_* = (L+R)/2, is equivalent to fluctuating asymmetry (FA = |L-R|/*m_i_*)[38] averaged over each group of 50 isogenic flies in the *P582i* experiment (Fig. 1). Microenvironmental variation (*V_e_ among*) was defined as the unsigned deviation of L+R traits of each fly from its clone mean (*m_c_*). Because most flies were symmetric for left and right bristle number, measures of *V_e_ within* for all bristle traits were highly discrete. Allelic effects for *V_e_ within* were compared using the Mantel-Haensel (M-H) test of differences in the size of symmetrical and non-symmetrical classes between groups of *P582i* and control (*Sami*) flies. The trait distributions of the most invariant bristle types, SC, TH and OR were also highly discrete, and were analyzed using non-parametric M-H tests. However, for most comparisons, ANOVA gave qualitatively similar results. WC was fully continuous and testable only by ANOVA. Asterisks indicate P-values significant after correction for multiple comparisons.

### ‘Extrinsic evolvability’, selection response

**Figure 4:**
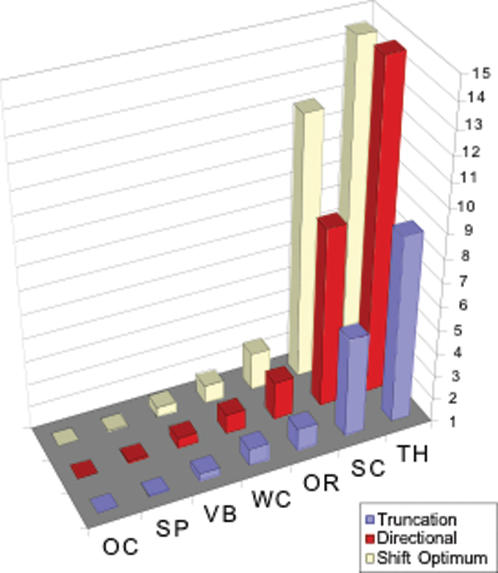
Hsp90-buffered variation contributed to predicted selection responses. Fold-increase in the predicted response to truncation, directional or stabilizing selection of the ‘populations’ of 9 *RI* line genotypes in the *P582i* mutant relative to equivalent ‘populations’ of genotypes in the *Sami* control flies (Table 5).

Since Hsp90 controlled sources of environmental variation (*V_e_*) and genetic variation manifest as strain-dependent effects (*V_GXG_*), we wondered whether quantitative changes controlled by Hsp90, if favorable, could respond effectively to selection. For example, non-genetic sources of variation such as *V_e_ within* and *V_e_ among* cannot contribute to evolution, and should impede the response to many types of selection. To predict Hsp90 effects on ‘extrinsic evolvability’ [40], [41], we calculated the expected selection response for each of the 7 traits in the synthetic “population” of 9 *RI* line backgrounds, using each traits' total phenotypic (*V_P_*) and genetic (*V_G_*) components of variation and under any of three models of selection. For 6 of the 7 traits, all estimates of evolvability were higher in *P582i* mutant population than in the wild-type control population (Table 5). However, while this suggested WC and OR were 1.5 to 2 fold more likely to respond to selection, much more striking, previously invariant SC and TH bristle numbers were potentially as much as 14-fold more evolvable under some models of selection (Fig. 4). Despite the overall increase in random and purely non-genetic effects among the Hsp90 mutant flies, when previously invariable SC or TH bristle numbers were destabilized, they became simultaneously sensitized to the effects of multiple types of variation and their predicted responses to selection were also dramatically increased.

**Table 5:**
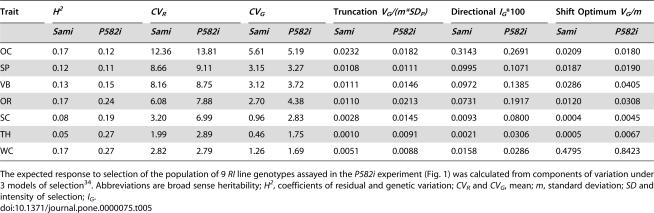
Estimates of ‘extrinsic evolvability’ were increased in isogenic backgrounds mutant for Hsp90 (*P582i*).

Trait	*H^2^*		*CV_R_*		*CV_G_*		Truncation V_G_/(m*SD_P_)		Directional I_G_*100		Shift Optimum *V_G_/m*	
	*Sami*	*P582i*	*Sami*	*P582i*	*Sami*	*P582i*	*Sami*	*P582i*	*Sami*	*P582i*	*Sami*	*P582i*
OC	0.17	0.12	12.36	13.81	5.61	5.19	0.0232	0.0182	0.3143	0.2691	0.0209	0.0180
SP	0.12	0.11	8.66	9.11	3.15	3.27	0.0108	0.0111	0.0995	0.1071	0.0187	0.0190
VB	0.13	0.15	8.16	8.75	3.12	3.72	0.0111	0.0146	0.0972	0.1385	0.0286	0.0405
OR	0.17	0.24	6.08	7.88	2.70	4.38	0.0110	0.0213	0.0731	0.1917	0.0120	0.0308
SC	0.08	0.19	3.20	6.99	0.96	2.83	0.0028	0.0145	0.0093	0.0800	0.0004	0.0045
TH	0.05	0.27	1.99	2.89	0.46	1.75	0.0010	0.0091	0.0021	0.0306	0.0005	0.0067
WC	0.17	0.27	2.82	2.79	1.26	1.69	0.0051	0.0088	0.0158	0.0286	0.4795	0.8423

The expected response to selection of the population of 9 *RI* line genotypes assayed in the *P582i* experiment (Fig. 1) was calculated from components of variation under 3 models of selection^34^. Abbreviations are broad sense heritability; *H^2^*, coefficients of residual and genetic variation; *CV_R_* and *CV_G_*, mean; *m*, standard deviation; *SD* and intensity of selection; *I_G_*.

Dominance variation, and variation resulting from the possible sensitivity of Hsp90-mutant flies to shared environmental effects would have confounded our estimates of extrinsic evolvability. To test the effect of Hsp90 on evolvability directly, we measured the response of SC bristle number to artificial selection. To get flies with abnormal SC bristles we raised a wild-type lab strain of flies (*Canton-S*) for one generation on media containing the Hsp90 inhibitor geldanamycin (GA). Parents with varying numbers of SC bristles were selected and crossed in groups of increasing mean SC bristle number. Their offspring were split during early larval stages into replicates raised on media with or without GA. Not surprisingly, without inhibitor the averaged SC phenotype of control flies followed the increasing SC bristle numbers of their parents, showing that although SC bristle numbers do not normally vary and could not be selected, populations still carry cryptic variation for this trait [26]. Realized heritability (*h^2^*), a measure of additive genetic variation (*V_A_*) contributing to the selection response, was reflected in the positive regression of offspring onto parental bristle number (Table 6). An abundance of *V_A_* affecting even normally-invariant or canalized traits is suggested by the ubiquity of “genetic background effects” and by the abundance of specific enhancers and suppressors of major developmental mutations [42]. In early experiments used to demonstrate canalization, mutations or environmental perturbations initially reveal abnormalities and specifically document cryptic variation contributing to selection response [26], [43], [44]. Indeed, extensive work by Rendel provides rigorous evidence for cryptic genetic variation and buffering of SC variation at even numbers of bristles by successive developmental thresholds [26].

**Table 6:**
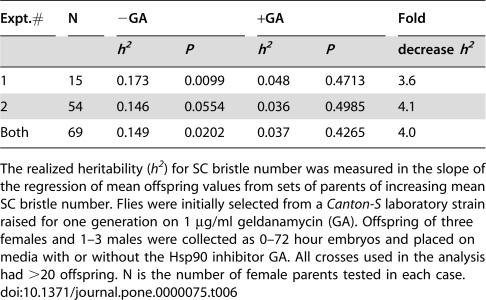
Hsp90 controlled SC realized heritability and selection response.

Expt.#	N	−GA		+GA		Fold
		*h^2^*	*P*	*h^2^*	*P*	decrease *h^2^*
1	15	0.173	0.0099	0.048	0.4713	3.6
2	54	0.146	0.0554	0.036	0.4985	4.1
Both	69	0.149	0.0202	0.037	0.4265	4.0

The realized heritability (*h^2^*) for SC bristle number was measured in the slope of the regression of mean offspring values from sets of parents of increasing mean SC bristle number. Flies were initially selected from a *Canton-S* laboratory strain raised for one generation on 1 µg/ml geldanamycin (GA). Offspring of three females and 1–3 males were collected as 0–72 hour embryos and placed on media with or without the Hsp90 inhibitor GA. All crosses used in the analysis had >20 offspring. N is the number of female parents tested in each case.

Contrary to the simple expectation that reduction of Hsp90 by GA would increase the heritability and response to selection of SC bristle number, we found that in our inbred *Canton-S* stock, the selection response of offspring to increasing parental SC bristle numbers was completely abolished in the replicates raised on the Hsp90 inhibitor. The slope of the response and *h^2^* were not different than 0 (Table 6). This surprising result was not simply an artifact of increased non-genetic variation among flies raised on GA. We found that in the *Canton-S* genetic background, GA decreased rather than increased phenotypic variation in SC (*CV_−GA_* = 0.053, *CV_+GA_* = 0.035; Bartlette's *P* = 0.0057, Levine's *P* = 0.4083). Even more surprising, the offspring of parents with 4 SC bristles had a higher number of SC bristes, regardless of GA treatment. Alleles causing extra SC bristles were therefore already segregating in our *Canton-S* stock. Indeed, when we examined untreated *Canton-S* flies, 9/197 (∼4.6%) had extra SC bristles due to variation that likely accumulated from the combined effects of loss of selective pressure, inbreeding and drift under standard conditions of laboratory propagation in vials.

Variation for abnormal SC was not restricted to potentially deleterious alleles accumulated in the unnatural environment of the lab. A strain dependence for the direction of Hsp90 effects on SC was also seen in one of the wild *RI* line backgrounds. In the *RI-27* heterozygotes, the proportion of flies with abnormal SC numbers (in this case, 3 SC) was also higher in the Hsp90 wild-type background (Fig. 3; *Sami*; 10% abnormal; *CV* = 0.078). Similarly, when Hsp90 was mutant in *RI-27* heterozygotes, the number of flies with abnormalities, and all forms of SC bristle variation, were simultaneously decreased (*P582i*; 2% abnormal; *CV* = 0.035; Levine's *P* = 0.0005). Interestingly, contradictory and strain-dependent effects on trait variability were described in classic studies of Drosophila wing vein canalization [44]. Heat shock during a particular window of development normally produced flies with a crossveinless phenotype. However, rare strains have a high frequency of flies expressing crossveinless abnormalities, which were inexplicably reduced by heat treatment.

### Threshold model of Hsp90-buffered variation

Well-characterized mechanisms of bristle development together with known Hsp90 biochemistry and standard quantitative genetic theory provide a coherent model encompassing the confusing, contradictory and strain dependent effects of Hsp90 within classical theory of threshold traits. An across-the-boards reduction of signal transduction resulting from Hsp90 mutation (or heat stress) would exacerbate the phenotypic effects of signaling weakened by the chance accumulation of reduced-function polymorphisms. But the same Hsp90-dependent reduction in signaling could also bring overactive signaling back down into the normal ranges, ameliorating the effects of a chance accumulation of activating polymorphisms. For example, in inbred lab strains, depending on genetic context the same Hsp90 alleles both enhance heterozygous loss-of-function mutations in other pathway components, exposing developmental phenotypes [5], and suppress over-expression phenotypes of dominant activating mutations in the same pathways, restoring normal phenotypes [34]. Our data now suggest Hsp90 has similar effects on natural variation in the developmental pathways underlying quantitative traits.

**Figure 5:**
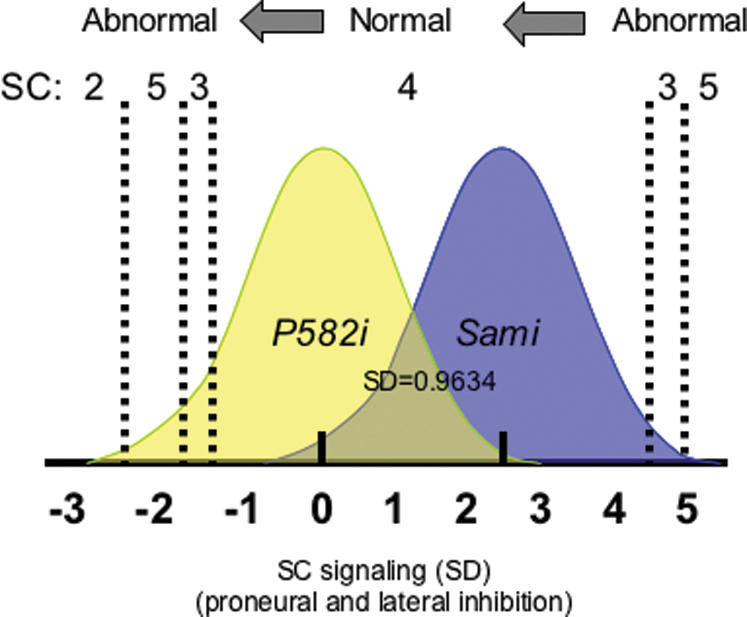
Upper and lower thresholds control SC bristle number. A threshold trait model was applied the effect of Hsp90 on signalling pathways underlying SC bristle development with assumptions based on current understanding of bristle development and Hsp90 function [26], [46]. The positions of upper and lower thresholds were estimated for *P582i* and *Sami* populations, which differed by the presence of wild-type (*Sami*) or heterozygous mutant (*P582i*) levels of Hsp90 in otherwise nearly identical genetic backgrounds (N = 450 flies each). A consistent, but arbitrary, standard deviation (SD) scale was inferred from the frequency of abnormal flies in the P582i background, which had the highest frequencies of abnormal flies. SC was canalized at 4 over a range of from 5 1/2 to 6 1/2 SD. On the same scale, we estimate that the *P582i* mutation decreased the strength of proneural and inhibitory bristle development pathways, shifting signalling across the population >2.5 SD to the left relative to fixed upper and lower thresholds.

Classical threshold trait models posit unseen distributions of cryptic genetic and environmental effects contributing to the probability of expressing discrete traits such as deformity or disease. To compare unseen distributions of continuous variation in the strength of SC bristle signaling in Hsp90 mutant and wild-type populations of *RI* line genotypes, we modeled SC as a classical threshold trait. Using the observed frequencies of flies with abnormal SC bristle numbers (SC = 2, 3, 5) in *P582i* mutant and *Sami* populations and a set of simple assumptions consistent with Hsp90 biochemistry and current understanding of bristle development, we determined inferred distributions of liability for abnormal SC numbers in each population. For example, we reasoned that a reduction in SC bristles could be caused by either abnormally low levels of proneural signaling or by abnormally high levels of inhibitory signaling [24], [45]. As shown in Figure 5, under the simple assumptions of the threshold model, the distribution of hidden variation in the strength of SC signaling through combined proneural and lateral inhibition pathways (liability for abnormal bristle numbers) was shifted more than 2 1/2 SD units to the left in the *P582i* mutant flies. Indeed, threshold models featuring hidden distributions of genetic and/or environmental effects are the only way to describe the inheritance of highly discrete polygenic traits within the framework of standard quantitative genetics [26], [46], [47]. As shown here, these threshold models resolve the apparently contradictory effects of Hsp90 buffering (or heat stress [44]) on discrete traits such as SC bristle number and provide a coherent theoretical basis for Hsp90 effects on phenotypic variation.

## Discussion

### Is Hsp90 a “canalization gene”?

The remarkable selectivity of Hsp90 for invariant (or canalized) features of both qualitative and quantitative traits is a surprising and previously unrecognized property of Hsp90 buffering, providing an organic distinction between the biological control of variation of normally invariant quantitative and qualitative traits on the one hand, and control of more naturally variable quantitative (or life history [48]) traits on the other. This distinction transcends an historical dichotomy between quantitative and qualitative traits, and is consistent with Waddington's original restriction of canalized traits to those with discrete developmental outputs [27] (e.g. qualitative morphologies and invariant (discrete) quantitative traits such as SC and TH [26]). Specific genetic mechanisms of canalization are implicit in the definition of canalization as an evolved feature of development. But is Hsp90 a “canalization gene” as has been suggested [16], [18]. Did the Hsp90 chaperone system evolve to prevent the expression of abnormal phenotypes?

Stabilizing selection for an optimum intermediate phenotype would select against individuals with reduced genetic and environmental buffering. However, despite the fact that Hsp90 buffers developmental variation, the idea that Hsp90 chaperone and co-chaperone interactions with nearly 200 different signalling proteins evolved because of selection for canalization of development is hard to understand based on any known molecular mechanism, and difficult to reconcile with the trait-specificity and contradictory genetic background effects of Hsp90 we report here. The specificity of Hsp90 buffering for the most invariant (or canalized) features is suggestive of a general canalization gene, but for some traits in certain genetic backgrounds Hsp90 supported rather than masked abnormal phenotypes, which were more normal and less variable when Hsp90 was mutant or reduced by drug. And at least one highly invariant trait, left-right asymmetry in wing area, was tightly buffered independent of Hsp90.

It is also difficult to imagine how evolutionary recruitment of Hsp90 to particular components of a wide range of signalling pathways would increase developmental robustness or reduce variability. Hsp90 almost certainly originated as a chaperone for general protein folding and stability, and it seems much more likely it was later co-opted for the benefits of regulated signal transduction rather than because of selection for developmental stability. Hsp90 stabilization of immature and inactive signal transducers would allow previously constitutive signalling proteins to be held in late stage folding by the Hsp90 chaperone complex, poised until association with ligand or other proteins complete their maturation, folding and activation. Indeed, conformational changes associated with activation usually stabilize and “mature” Hsp90 targets, completing their folding [3]. It seems much more likely that robustness against genetic and environmental variation emerge or evolve on a trait-by-trait basis. Many genes buffer genetic variation through extensive epistatic interactions [49]; it is long established that the phenotypes of developmental mutations are more variable than the wild-type [42]. Hsp90 is repeatedly identified as a component of these same signalling pathways by genetic interaction screens. As a player in multiple pathways controlling many morphological features, one could predict that Hsp90 would control the variability of many different adult phenotypes, which become more variable as Hsp90 (and Hsp90 target function) is reduced.

Development can be thought of as a series of discrete decisions, integrating continuous input from numerous sources and translating them into all-or-none commitments. This digitization may be a key to the high fidelity and robustness of development in the face of noisy inputs from random environmental disturbances and genetic variation. As noted by Waddington, cells and tissues rarely get stuck in intermediate forms, but choose between alternate types suggestive of all-or-none switches [27]. Robustness against genetic and environmental perturbation is not a property of any single gene, but is an emergent property resulting from the topology of connections between interacting genes. Genetic circuit properties that convert continuous responses into all-or-none events are increasingly well understood. For example, biological switches have been modelled mathematically [50], [51], discussed theoretically [52] and, more recently, synthesized in bacteria based on first principles [53], [54]. Mitogen activated protein kinase (MAPK) pathways are Hsp90-dependent, and their non-linear kinetics are particularly well-studied [51], [52], [55]. The MAPK phosphorelay system consists of three kinases in a linear pathway. Modelling MAPK pathway upstream inputs and downstream response using basic Michaelis-Menton kinetics describes increasing non-linearity (Hill coefficient) of the downstream response with successive addition of intervening kinases [55]. Indeed, inflection points of sigmoidal responses of phenotype to continuous variation in the strength of underlying signals are a form of threshold.

**Figure 6:**
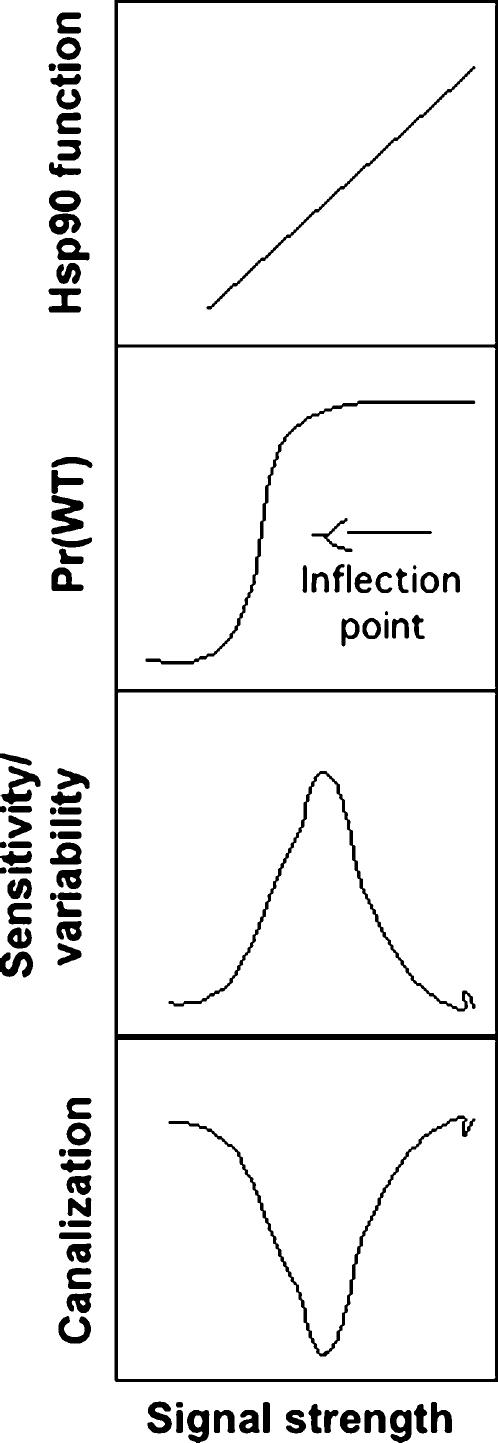
Model for biological control of canalization and evolvability. In any individual the strength of signaling through Hsp90 target pathways is directly proportional to the level of Hsp90 function (top panel), but the phenotypic effects of changes in signaling strength are often highly nonlinear, creating thresholds (the inflection point, second from top). A simple consequence of steep non-linearity is that sensitivity to all kinds of perturbation and stochastic effects is expected to be highest at the inflection point (second panel from bottom). This simple relationship between sensitivity to variation and the steepness of the signaling-phenotype relationship at the inflection point, the inverse of canalization (bottom panel), suggests why all variation increases in concert for individuals and/or populations near trait thresholds.

We suggest that a defining feature of many Hsp90-dependent pathways may be they govern switch-like developmental decisions such as cell-fate or tissue-type specification. By definition, these non-linear developmental responses are insensitive (canalized) a wide range of input (Fig. 6). Selective pressure to buffer morphological variability in response to continuously varying developmental signals would favour the evolution of non-linearity and trait thresholds, which could occur relatively simply by chance addition of regulatory or positive feedback connections. But regardless of whether developmental stability and the digitization of development are selected adaptations, emerge as unselected by-products of genetic redundancy or are an unavoidable feature of the architecture of developmental networks, non-linearity of Hsp90-dependent pathways such as MAPK would both enable and explain Hsp90 control of phenotypic variability and canalization.

## Materials and Methods

### Strain construction and inhibitor study

The strains used in this study and geldanamycin treatments have been described [19]. To isolate the allelic effects of Hsp90 on quantitative trait variation independent of the effects of differing genetic backgrounds in mutant and control lines, we constructed highly isogenic Hsp90 mutant (*P582i*) and control (*Sami*) strains. A marked P-element allele of Hsp90 (*P582*) was introgressed into the extremely isogenic background *Samarkand* (*Sam*; kindly provided by T.F.C. Mackay). *Sam* was completely homozygous at nearly 100 350–500 bp sites sampled randomly across the 2^nd^ and 3^rd^ chromosomes (>30 Kb; S.R. unpublished). Based on more than 35 generations of backcrossing and homogenization, we estimated that less than 5 cM of the original *P582* background would flank the P-element insertion site. To produce matched sets of mutant and control flies, heterozygous *P582i* females were crossed to males from 9 highly inbred (>95% homozygous) Raleigh Inbred (*RI*) lines representing genotypes sampled at random from a wild population [56]. To control environmental variation, the F_1_ progeny were picked as 2nd instar larvae and raised in 5 replicated density, temperature (25°C.) and humidity controlled vials, after which 10 males per vial were selected at random and scored for bristle and wing features.

To study the effects of additional, unmarked alleles of Hsp90, entire Hsp90 mutant 3^rd^ chromosomes were crossed into the standard *Sam* background with over 10 generations of backcrossing [19]. Virgins heterozygous for either dominant negative (*9J1*) or null (*P582*) alleles balanced over *TM6B* in the *Sam* background (*Sam1;Sam2;9J1/TM6B or Sam1;Sam2;P582/TM6B*) were crossed to males from each *RI* line background. 5 flies of each sex were scored from each of 5 density, temperature, and humidity controlled replicate vials as described above. The TM6B controls and Hsp90 mutant sibs were from the same vials, reducing environmental and maternal effects. The experiment to test the effect of the Sam 3rd chromosome against each *RI* line background (*Sam1;Sam2;Sam3* virgins X *RI* line males) was done separately. As TM6B contains the dominant Humeral (Hu) mutation [32], which increases humeral TH bristle numbers, TH-HU was used for comparison of TH bristles between TM6B and the other genotypes, indicating that TH was scored only for the remaining 18 non-humeral TH bristle types.

In experiments testing the effects of reduced Hsp90 on wild-type backgrounds Hsp90 was inhibited by geldanamycin. An outbred laboratory population of *Canton-S* flies, which had been maintained for several years in population cages to maintain a high effective population size (*N_e_*), was fed geldanamycin as 2^nd^ or 3^rd^ instar larvae. Adult flies were subsequently scored for quantitative wing and bristle traits. In the selection response experiment, a small and apparently inbred, laboratory stock of *Canton-S*. This strain had been maintained for many years under standard maintenance conditions in vials. Flies were raised on media containing 1 mg/ml geldanamycin. To measure the selection response, flies were collected as 0–72 hour embryos and split during early larval stages into replicates raised on media with or without geldanamycin, and adults were analyzed for SC bristle number.

### Selection of *dfe* flies

The estsablishment of the deformed-eye (*dfe*) selection has been described previously [6]. Briefly, among the F_1_ progeny of cross between a heterozygous Hsp90 mutants (*19F2*; [6]) and another lab strain we found a single heterozygous male (*19F2*/+) with eye deformity. That male was selected and crossed to 3–4 related, heterozygous females from the same *F_1_* population, yielding several more flies with eye deformities in the *F_2_*. By generation 4 numbers of dfe flies to found 3 related, but independent selection lines (high and low eye penetrance lines HE1–3 and LE1–3 [6]). Each generation, *dfe* flies were selected and crossed together and a similar number of related normal sibs were selected and crossed together to found high and low selection lines. With successive generations the *dfe* penetrance (fraction affect progeny) increased among the high lines, to the point that sufficient *dfe* polymorphisms were present that the trait no longer depended on Hsp90 reduction. The *19F2* allele, which was apparently deleterious even in heterozygous state, was coincidently lost. By generation 15, none of the flies from either high or low lines carried an Hsp90 mutation [6]. Generation times were kept long in these lines by successive passaging of flies. Fitness tests were initiated on stocks isogenized from flies in generation *F_42_* of selection.

### Data analysis

Bristle traits SC, TH, SP, OR, OC and VB were scored on the left and right sides of each fly, after which wings were removed and photographed. Wing centroid (WC) is an overall measure of size. WC was determined from the exact positions of eight junctions of longitudinal veins with crossveins or wing margins. Trait means, standard deviations and coefficients of variation (*CV* = standard deviation/mean) were used to compare phenotypic variation among flies across all 9 *RI* lines between traits. Since equal numbers of flies from each cross and genotype were scored, we followed a balanced design for data analysis. For the *P582i*/*RI* experiment, Hsp90 mutant effects were compared in matched sets of sibs from the same vial/maternal environment but carrying a wild-type allele of Hsp90. For the *P582* and *9J1* experiment, Hsp90 mutant effects were compared to wild-type alleles of Hsp90 on the *TM6B* balancer chromosome or the *Sam* 3^rd^ chromosome. In this experiment the genetic backgrounds of the Hsp90 mutants and *Sam* controls differed by nearly 1/3^rd^ of their genomes.

To measure the Hsp90-genetic background interaction effects, weighted ANOVAs were used to control for heteroscadacity, particularly in the distributions of the more discrete bristle types (SAS software, SAS Institute, Raleigh, NC). The effect of the *P582i* and *Sami* alleles of Hsp90, *RI* lines and their interaction on wing size and bristle traits were compared among the groups of 50 isogenic flies in each of the 9 *RI* line backgrounds. P-values were corrected for multiple comparisons using the Bonferroni correction.

Developmental noise and micro-environmental variation were analyzed in the *P582i* experiment. Developmental noise (*V_e_ within*), defined for each fly as the average unsigned deviation of left and right features from their mean within each individual or (|L-*m_i_*|/*m_i_*+|R-*m_i_*|/*m_i_*)/2 where *m_i_* = (L+R)/2, is equivalent to fluctuating asymmetry (FA = |L-R|/*m_i_*) averaged over the mean values for each group of 50 isogenic flies from each *RI* line background. Microenvironmental variation (*V_e_ among*) was defined as the unsigned deviation of L+R traits of each fly from its clone mean (*m_c_*). Because most flies were symmetric for left and right bristle number, measures of *V_e_ within* for all bristle traits were highly discrete. Allelic effects for *V_e_ within* were compared using the Mantel-Haensel (M-H) test of differences in the size of symmetrical and non-symmetrical classes between groups of *P582i* and control (*Sami*) flies. The trait distributions of the most invariant bristle types, SC, TH and OR were also highly discrete, and were analyzed using non-parametric M-H tests. However, for most comparisons, ANOVA gave qualitatively similar results. WC was fully continuous and testable only by ANOVA. P-values were corrected for multiple comparisons.

The effect of wild-type and mutant alleles of Hsp90 on the expected response to selection of the population of 9 *RI* line genotypes was approximated using observed components of genetic and environmental variation in the *P582i* experiment, and according to methods described by Houle [41]. Broad sense heritability (*H^2^*) and total genetic variation (*V_G_*) were substituted for realized heritability (*h^2^*) in these approximations, would have unpredictable effects on the exact approximations obtained. However, the size and consistency of the effects of Hsp90 allele, and an effect of Hsp90 on additive variation and realized heritability demonstrated by response to artificial selection confirm the overall conclusions. The effect of Hsp90 on *h^2^* and *V_A_* for qualitative traits was confirmed previously [6], and an effect of Hsp90 on *h^2^* and *V_A_* of SC was calculated from the slope of the regression of the mean offspring values from sets of parents of increasing mean scutellar bristle number using offspring estimates of mean SC from crosses which had >20 F_1_ progeny.

### Threshold model

Thresholds for expression of abnormal SC bristle numbers were modeled based on known Hsp90 genetics and biochemistry, bristle development pathways and a set of simple assumptions of threshold trait models:

1) Loss-of-function mutations are generally recessive to wild-type alleles. We followed Rendel [25] in assuming that wild-type populations are closer to upper thresholds. In our case, the upper threshold was explicitly defined by line *RI-27*. (By contrast with any other *RI* line, *RI-27* had significantly more abnormal flies in the *Sami* background than in *P582i* mutants.) For nearly every known Hsp90 target, reduction of Hsp90 decreases the activity of its substrate proteins [5], [53]. For these reasons, we placed the *P582i* distribution to the left of the *Sami* distribution, indicating that the *P582i* population had reduced overall levels of signaling.

2) Bristle patterning in Drosophila is accomplished by a complex network of proneural signalling and lateral inhibition, with opposing effects on bristle number [54]. Decreased proneural function beyond a lower threshold reduces bristle number, decreased lateral inhibition increases bristle number, and over-activation of either process would have the opposite effect. Liability for abnormal bristles is a function combining opposing signals through these pathways.

4) To put the distributions on the same scale required that we assume that the SD of both *Sami* and *P582i* distributions were equal [37]. As shown by our data this is not a correct assumption. However, if we included the increased variance of the *P582i* population in the model, the calculated shift in distributions would be greater than the shift seen here. Therefore we have estimated a minimum bound for Hsp90 effects.

5) The order of thresholds was determined by the relative frequencies of each SC bristle class (2,3,4,5) initially on a standard normal distribution (m = 0, SD = 1) with the most likely classes being closest to normal (4SC).

6) We assumed that the means of each *RI* line distribution in *Sami* would be shifted left by similar amounts by the *P582i* mutant. Inverse probability densities were calculated using http://davidmlane.com/hyperstat/z_table.html. In the *Sami* background most *RI* lines had no abnormal flies; 5/450 flies had 3 SC (0.0111; all in line *RI-27*), and 3/450 flies had 5 SC (0.0067). In the *P582i* population across 9 *RI* lines 19/450 flies (0.0422) had 3 SC bristles, 12/450 had 5 SC (0.0267) and 3/450 had 2 SC (0.0067).

The order of thresholds, determined by the relative frequencies of each SC bristle class (2,3,4,5) was initially based on standard normal distributions (m = 0, SD = 1) with the most likely classes being closest to normal (4SC). The lowest (rightmost) threshold was defined with 2 at the rightmost position next to 5 (2|5; −2.473), the second lowest (5|3; −1.833), and the third threshold for the most frequent aberrant bristle class (3) was adjacent to normal (3|4; −1.435). Using methods described by Wright [46], we arbitrarily converted the distance between the 2|5 and 3|4 thresholds to 1 SD unit by converting the SD of the *P582i* distribution to (1/distance between old thresholds) and calculating the positions of the new, SD-normalized thresholds (the products of the previous thresholds and the new SD = 0.9634) [37]. We next placed the wild-type (*Sami*) distribution on the same scale, with the mean of the lower distribution (*P582i*) still arbitrarily set to 0. The mean of the *Sami* distribution (N = 450) was determined using three different methods based on different assumptions. First, the 3 *RI* line/*Sami* distributions (N = 50) that had abnormal flies presumably beyond an upper threshold (in *Sami*) and below the lower threshold (in *P582i*). We determined the expected means of each *RI* line distribution in *Sami* and *P582i* based on frequencies of normal (4SC) flies in *Sami* on the lower 3|4 threshold and in *P582i* on the upper 4|3 threshold. We then calculated on the SD scale, the average shift left of each distribution resulting from the *P582i* allele. Averaging the means of the 9 *RI* line/*Sami* distributions set the mean shown for the global *Sami* distribution (N = 450) at 2.47, allowing upper (rightmost) thresholds for the *Sami* distribution to be calculated by probit analysis (4|3, 4.495; 3|5, 4.554). Three different methods for determining the upper threshold geldanamycin gave similar results: from 2 (assuming that the frequency of abnormal flies beyond the lower threshold in the *Sami* distribution was<0.001) to 2.2 assuming that only the abnormal flies in *RI-27* were beyond the upper threshold in the *Sami* background), to 2.47 as determined above.
